# Hepatitis A and E Viruses Are Important Agents of Acute Severe Hepatitis in Asia: A Narrative Review

**DOI:** 10.3390/pathogens14050454

**Published:** 2025-05-06

**Authors:** Reina Sasaki-Tanaka, Tatsuo Kanda, Takeshi Yokoo, Hiroyuki Abe, Kazunao Hayashi, Akira Sakamaki, Hiroteru Kamimura, Shuji Terai

**Affiliations:** 1 Division of Gastroenterology and Hepatology, Graduate School of Medical and Dental Sciences, Niigata University, Niigata 951-8520, Japan; reina_sasaki_0925@yahoo.co.jp (R.S.-T.); t-yokoo@med.niigata-u.ac.jp (T.Y.); hiroyukiabe@med.niigata-u.ac.jp (H.A.); khayashi@med.niigata-u.ac.jp (K.H.); saka-a@med.niigata-u.ac.jp (A.S.); hiroteruk@med.niigata-u.ac.jp (H.K.); terais@med.niigata-u.ac.jp (S.T.); 2 Division of Gastroenterology and Hepatology, Uonuma Institute of Community Medicine, Niigata University Medical and Dental Hospital, Uonuma Kikan Hospital, Minami-Uonuma, Niigata 949-7302, Japan

**Keywords:** Asia, genotype, hepatitis A virus, hepatitis E virus, vaccine

## Abstract

Acute-on-chronic liver failure (ACLF) and acute liver failure (ALF) are severe hepatitis that occur in patients with and without chronic liver diseases and/or cirrhosis, respectively, and both often result in death. Hepatitis A virus (HAV) and hepatitis E virus (HEV) infection can cause these severe conditions. We reviewed the role of HAV and HEV, which infect humans through the fecal–oral route, in ALF and ACLF in Asian countries. This narrative review was the derived from a traditional non-systematic review. Hepatitis A should be recognized as one of the sexually transmitted infections, especially among men who have sex with men. HAV genotype IIIA infection seems to present a more severe clinical manifestation. Acute HEV-1 infection is associated with ALF in pregnant women in India. HEV-4, rather than HEV-3, was found in severe hepatitis in Japan. HEV also plays a role as a cause of acute insult and/or chronic liver disease in immunocompromised patients with ACLF. Further studies are needed for the development of vaccines and antivirals against HAV and HEV infections. Despite the limitations of the recording of cases and the extent of specific vaccinations, multidisciplinary cooperation, involving hepatologists, virologists, experts in public health, etc., may improve the treatment of HAV and HEV infection.

## 1. Introduction

Acute-on-chronic liver failure (ACLF) and acute liver failure (ALF) are severe conditions that occur in patients with and without chronic liver diseases and/or cirrhosis, respectively. Both ACLF and ALF are associated with high mortality rates unless liver transplantation is performed [1,2,3]. Viral infection is a common cause of ALF in Asian countries [4,5,6,7], and viral hepatitis is one of the major causes of ACLF in Asia [8,9,10].

The global burden and trends of acute viral hepatitis among children and adolescents from 1999 to 2019 demonstrate that the high-incidence regions include Sub-Saharan Africa, Oceania, South Asia, and Central Asia, with India, Pakistan, and Nigeria facing the greatest burden, and that the leading cause is hepatitis A virus (HAV), followed by hepatitis E virus (HEV), hepatitis B virus (HBV), and hepatitis C virus (HCV) infection [11].

HAV infection causes acute hepatitis, occasionally leading to ALF and ACLF [12], as well as extrahepatic manifestations. HEV infection causes acute hepatitis, including ALF and ACLF, chronic hepatitis (especially in compromised hosts), and various extrahepatic manifestations [13,14,15]. Acute HEV infection may also lead to ALF in women during pregnancy [13].

It has recently become easier to treat patients with HBV or HCV infection due to the development of nucleoside/nucleotide analogues [16,17,18] and direct-acting antivirals (DAAs) [19], although there are several issues still to be addressed in this area [20,21,22].

Here, we review the roles played by HAV and HEV infection in ALF and ACLF in Asia. We also discuss the present situation regarding the development of specific treatment and preventive methods, including vaccines, for HAV and HEV infection.

## 2. Acute Liver Failure (ALF) and Acute-on-Chronic Liver Failure (ACLF)

ALF—or fulminant hepatic failure—occurs in patients without pre-existing liver diseases. It is a life-threatening disease characterized by severe liver damage, with coagulopathy, hepatic encephalopathy, and high mortality [23]. ALF is defined according to the time interval between the development of symptoms and the onset of hepatic encephalopathy [23,24].

The Asian Pacific Association for the Study of the Liver (APASL) ACLF Research Consortium (AARC) for APASL ACLF Working Party has defined ACLF as acute hepatic insult manifesting as jaundice and coagulopathy leading to complications within 4 weeks (defined by ascites and/or encephalopathy) in patients with previously diagnosed or undiagnosed chronic liver disease, and this condition is associated with high mortality [1].

In Japan, patients showing 40% or less of the standardized prothrombin time value or having an INR of 1.5 or greater caused by severe liver damage within 8 weeks of onset of symptoms are diagnosed as having ALF [25,26,27]. Gimson et al. defined patients in whom hepatic encephalopathy occurs between 8 and 24 weeks after the first symptoms of liver disease as suffering from late-onset hepatic failure (LOHF) [28].

In Japan, ACLF has been defined as a patient with cirrhosis and a Child–Pugh score of 5–9 in whom there is a deterioration in liver function (serum bilirubin level equal to or greater than 5.0 mg/dL and prothrombin time value equal to or less than 40% of the standardized values and/or international normalization rate equal to or greater than 1.5) caused by severe liver damage developing within 28 days after acute insult (e.g., alcohol abuse, bacterial infection, gastrointestinal bleeding, or the exacerbation of underlying liver diseases) [29].

Thus, although there are various definitions, ACLF and ALF may be understood as severe forms of acute hepatitis in patients who may or may not suffer from chronic liver disease. As ALF and ACLF patients also have poor prognoses without liver transplantation, it is very important to diagnose their etiologic agents.

It is now widely known that ALF is caused by various viral infections, drugs, autoimmune hepatitis, and other factors. In Japan, among a total of 1554 and 49 patients with ALF and LOHF, respectively, who were seen between 2010 and 2015, viral infection, drug-induced liver injury, and autoimmune hepatitis were observed in 28.9% (463/1603)—including HAV (6.4% (103/1603)), HBV (18.8% (302/1603)), HCV (0.7% (12/1603)), and HEV (1.4% (23/1603))—15.5% (248/1603), and 9.5% (152/1603) of patients, respectively [4].

The causes of ACLF are similar but also include excess alcohol intake [1,7,23]. In Asian countries, reactivation of HBV as an acute hepatic insult is the leading cause of ACLF [1]. In a Chinese study, HBV reactivation was observed in 60.9% (106/174) of patients with ACLF [30]. On the Indian subcontinent, superinfection with HEV is another important infectious event in ACLF cases [1]. In an Indian study, among ACLF patients, HAV, HEV, and both HAV and HEV infection were observed as acute insults in 27.3% (33/121), 66.1% (80/121), and 6.6% (8/121) of patients, respectively [8]; meanwhile, among those with chronic liver disease of ACLF, HBV, HCV infection, only alcohol and autoimmune conditions were observed in 30.6% (37/121), 4.1% (5/121), 10.7% (13/121) and 5.0% (6/121) of patients, respectively [8].

Acute viral hepatitis due to HAV/HEV is a common cause of ACLF in Asia–Pacific countries [1]. Therefore, we focused on HAV and HEV infections as causes of ALF and ACLF.

## 3. Hepatitis A Virus (HAV)

HAV is a non-enveloped RNA virus with a length of approximately 7.6 kb [12]. In general, HAV replicates mainly in the liver and is a non-cytopathic virus. However, HAV virions are egressed non-lytically from HAV-infected hepatocytes into blood vessels and bile canaliculus as quasi-enveloped virions (eHAV) cloaked in host membranes, similar to exosomes but lacking any HAV RNA genome-derived protein on the surface, such that eHAV can efficiently enter cells [12,31,32]. eHAV is resistant to neutralizing antibodies and is released across the canalicular membrane and stripped of membranes by bile acids acting as detergents within the proximal biliary canaliculus. A highly stable naked non-enveloped virion is thereby formed, which is shed in feces and optimized for transmission [33,34].

### 3.1. Diagnosis of Hepatitis A

In general, a diagnosis of hepatitis A is confirmed by a positive result for anti-HAV immunoglobulin M (IgM) antibodies [12]. Delayed anti-HAV IgM seroconversion as a positive result for anti-HAV IgM on a repeat test after an initially negative result was observed in 6.4% (38/595) patients with acute HAV infection in a Korean outbreak [35]. If a patient with a negative result for anti-HAV IgM antibody is suspected of having hepatitis A, then testing for the anti-HAV IgM antibody should be repeated during the 7 days following the first examination.

HAV is transmitted via the fecal–oral route through the consumption of HAV-contaminated food and water, as well as via person-to-person contact, such as through infection due to contact with household items that have come into contact with hepatitis A patients, sexual transmission, and foreign travel [12,36,37,38,39,40,41]. Data from the Japanese National Epidemiological Surveillance of Infectious Diseases program indicated that about 16.1% (141/874) individuals aged 60 years and older have anti-HAV antibodies, but only 1.2% (82/6993) of those aged below 60 years old have immunity; thus, almost all individuals younger than 60 years of age are susceptible to HAV infection [42]. As such, it should be noted that it is possible for HAV outbreaks to occur in developed and developing countries.

In Japan, an outbreak of acute HAV infection was observed between 2018 and 2020 [43,44,45,46,47]. These male-dominant HAV outbreaks were observed among human immunodeficiency virus (HIV)- and non-HIV-infected persons in Japan.

In Taiwan, an HAV infection outbreak was observed from June 2015 to September 2017 [48]. Compared with non-outbreak cases (*n* = 154), the outbreak cases (*n* = 145) tended to be male, have reported an HIV infection, presented or had a history of syphilis infection, had oral–anal sex within 2 months before symptom onset, and self-identified as men who have sex with men (MSM) (showed (64% (98/154) vs. 99% (144/145), *p* < 0.0001; 0% (0/154) vs. 52% (75/145), *p* < 0.0001; 0% (0/154) vs. 55% (80/145), *p* < 0.0001; 0% (0/154) vs. 30% (44/145), *p* < 0.0001; and 0% (0/98) vs. 60% (87/144), *p* < 0.0001; respectively) [48]. Hepatitis A should be recognized as one of the sexually transmitted infections, especially among MSM. Transfusion-transmitted HAV infection is rare but exists [49].

### 3.2. Symptoms of HAV Infection

In adults with acute HAV infection, jaundice, abdominal pain, appetite loss, nausea, vomiting, diarrhea, and hyperbilirubinemia peaking at 7–10 days after the onset of jaundice are typically observed [12]. Adults with acute HAV infection present with higher fever than do those with other types of acute viral hepatitis.

In children, HAV frequently causes an asymptomatic infection but rarely causes ALF. Among HAV infected children younger than 6 year old, 72% (13/18) are asymptomatic [50]. When HAV outbreak occurred in a childcare center located in a suburban area of Bangkok, Thailand, between November 2002 and February 2003, among children aged between 1 and 6 years with the anti-HAV IgM antibody, 91.5% (65/71) were asymptomatic, although 7.0% (5/71) children had acute clinical hepatitis [51].

### 3.3. HAV Genotypes and Clinical Manifestations

Although HAV—belonging to the *genus Hepatovirus* of the *Picornaviridae family*—has only one serotype, it has at least six genotypes (I to VI). It is well-known that three genotypes (I, II, and III) are of human origin [52]. It has been reported that the severity of HAV infection may not be associated with the nucleotide sequence of the HAV genotype-determining region in Japan, where the main HAV genotype is IA [53]. However, Korean patients with HAV genotype IIIA exhibit significantly higher aspartate aminotransferase levels, higher alanine aminotransferase levels, and lower platelet counts at baseline or peak/lowest laboratory data during the course compared with patients with HAV genotype IA [54]. Compared with HAV genotype IA infection, HAV genotype IIIA infection might present a more severe clinical manifestation [55,56].

Extrahepatic manifestations of HAV infection may also occur [35]. Prolonged cholestasis, severe jaundice, and recurrent hepatitis are occasionally observed [12]. Prolonged cholestasis was observed in 4.7% (28/595) Korean patients infected with HAV [35]. During the course of HAV infection, independent of liver disease severity, acute kidney injury and acute kidney failure may be observed, and some patients with hepatitis A require hemodialysis or hemodiafiltration. Among Korean patients infected with HAV, 1.5% (9/595) presented with acute kidney injury without ALF [35]. Attention should be paid to confusion with hepatorenal syndrome. Among Korean patients infected with HAV, 0.5% (3/595) presented with ALF, and mortality due to liver failure and spontaneous recovery was seen in one and two patients, respectively [35].

Hematological disorders, such as hemophagocytic syndrome, pure red cell aplasia, hemolytic anemia, and thrombotic thrombocytopenic purpura, along with neurological disorders, including Guillain-Barré syndrome and meningoencephalitis, are rarely observed [12]. Careful attention also should be paid to other extrahepatic manifestations.

### 3.4. Acute Severe Hepatitis A

It has been estimated that ALF progression occurs in 0.1–0.5% of patients with acute HAV infection. A Japanese study reported that the proportion of patients with fulminant hepatitis, ALF coma type with hepatic encephalopathy of grade 2 or higher, and/or LOHF caused by HAV infection were 6.4% (65/698), 2.9% (14/487), and 6.4% (103/1603) among patients with acute HAV infection who were seen between 1998 and 2003, between 2004 and 2009, and between 2010 and 2015, respectively [57]; however, the frequency of HAV infection is generally decreasing year by year.

In an Indian study, the most common cause of ALF was found to be HAV infection, which was recorded in 44.2% (81/183) of patients [58]. Also in India, ACLF due to HAV infection was observed in 27.2% (33/121) adult patients with cirrhosis [8]. Although the patients with HAV infection may suffer from malnutrition, further investigation of HAV strains, HAV nucleotide mutations, patients’ age, or other factors is needed. An HAV universal vaccination program may also be needed in India.

In Nepalese study, 92.7% (266/287) children with hepatitis in a tertiary care center were found to have hepatitis A. One child died due to complications, and the mortality rate was, therefore, 0.38% (1/266) among children with hepatitis [59]. In an Indian study, HAV infection was the most common cause of ACLF, occurring in 41.9% (13/31) of children [60]. It is possible that, in naïve populations, ALF is more common in adulthood than that in childhood even if the number of acute HAV infection cases are probably lower in adults than in infants. There are various host, viral, and other factors associated with severe HAV diseases; these have been described in a previous work by our group [12].

In developed countries such as Korea, the prevalence of hepatitis A was relatively low until the early 2000s due to improvements in the sanitary environment [41]. In 2009, 2010, and 2011, respectively, 58,651, 41,338, and 5492 cases of acute hepatitis A were reported. In 2019, a total of 10,083 cases of acute hepatitis A were reported for 7 months of the year by the Korea Center for Disease Control and Prevention despite HAV universal vaccination programs being started in May of 2015 [61]. A shift from HAV genotype IA to HAV genotype IIIA occurred in patients with hepatitis A in Korea.

It is important to note that HAV infection causes ALF in 2% (95% CI: 1–3) or 27% (95% CI: 13–43) of cases, respectively, in countries where HAV vaccination is performed or not [7]. It has been reported that after HAV infection, fecal shedding of HAV can last for months after the resolution of symptoms and that patients with this condition could be an important source of further local transmission [62,63].

### 3.5. Vaccines and Challenges Facing Developers of Anti-HAV Drugs that Prevent HAV Infection

Although the HAV vaccine has been shown to be effective in preventing HAV infection, HAV vaccination faces several challenges which remain to be solved. Three doses of HAV vaccine (Aimmugen^®^) for MSM living with HIV was found to be effective while two dose was less effective in non-HIV-infected people [64]. Four or more doses of HAV vaccine (Aimmugen^®^) may be effective when there is a need to ensure long-term immunity or if there is risk of prolonged exposure [65].

It is also important that anti-HAV drugs should be developed. Researchers have previously demonstrated the effectiveness of small interfering RNAs against HAV and HAV 3C cysteine protease inhibitors. The effectiveness of interferon-alfa, interferon-lambda-1, interferon-gamma, ribavirin, amantadine, and favipiravir have been shown. Several direct-acting antivirals (DAAs) against HAV or host-targeting agents (HTAs) have also been reported [66,67,68,69,70,71,72,73,74,75,76,77].

In previous works, we have also shown that the JAK2 inhibitor AZD1480, sirtuin inhibitor sirtinol, Japanese rice-koji miso, zinc chloride, zinc sulfate, and nicotinamide inhibit HAV replication. La protein, GRP78 (Bip), mitogen-activated protein kinase 3 (MAP2K3), and c-Jun are all critical targets of anti-HAV drugs [66,78,79,80].

A potential anti-HAV 3C protease inhibitor, Z10325150, was identified in a molecular docking study [67]. Artificial intelligence and machine learning methods could support the development of anti-HAV drugs [67,81]. It is important to develop and disseminate HAV vaccines at a lower cost and greater efficacy and to develop antivirals against HAV infection [12]. Useful recommendations and guidelines for the prevention of HAV infection have been produced in the United States [82,83], as well as in Asian countries [12]. However, further studies are still needed for the development of vaccines and antivirals against HAV infection.

In summary, the above review highlights the viral features of HAV, the present situation regarding HAV infection in representative Asian countries, and the development of anti-HAV drugs. It is important for medical researchers to be aware of the role of HAV as one of the causes of ALF and ACLF.

## 4. Hepatitis E Virus (HEV)

As HEV infection can potentially lead to ALF and ACLF, causing death or the need for liver transplantation, the prevention and treatment of HEV infection can be seen as a major health concern [14]. HEV infects humans through the fecal–oral route, causing acute hepatitis E. Hepatitis E also causes zoonosis and can lead to chronic infection in immunocompromised hosts [13,14,15].

The mammalian HEV genome is a single-stranded, positive-sense RNA with a length of approximately 7.2 kb [13]. HEV also exists in two distinct particle forms: HEV particles present in the bile and shed in the feces are classified as the membrane-unassociated form (non-enveloped HEV (neHEV)), while those in the bloodstream and culture supernatants are classified as the membrane-associated form (quasi-enveloped HEV (eHEV)) [13]. eHEV is coated with a lipid membrane that resembles the lipid membrane of exosomes [84]. Notably, HEV replicates in hepatocytes [85].

### 4.1. Diagnosis of Hepatitis E

In general, a diagnosis of hepatitis E is confirmed by a positive result for anti-HEV IgM antibodies. In Japan, anti-HEV IgA antibodies are also available. HEV infection is confirmed by a positive result for HEV RNA.

### 4.2. Symptoms of HEV Infection

Acute HEV infection rarely presents clinical symptoms in children [13]. In adults presenting with symptoms (such as flu-like myalgia, arthralgia, weakness, vomiting, jaundice, itching, uncolored stools, and dark urine), the incubation period ranges from 2 to 9 weeks [13].

### 4.3. HEV Genotypes and Clinical Manifestations

The various strains of the Hepeviridae family are classified as HEV-1 to HEV-8 within the *species Paslahepevirus balayani* [13,86]. HEV-1 and HEV-2 infect only humans and are related to HEV outbreaks in developing countries [87]. In addition, acute HEV-1 infection may lead to a greater incidence of ALF in pregnant women when compared with non-pregnant women and men in developing countries [14,88]. This may depend on the HEV genotype. HEV-3 and HEV-4 cause zoonosis, resulting in sporadic and autochthonous HEV infection in developed countries [87]. HEV-3 and HEV-4 are also major causes of chronic HEV infection in immunocompromised hosts and elderly persons [13,89]. In Japan, several studies have found HEV-4 in fulminant hepatitis E rather than HEV-3 [90,91,92]. HEV-5 and HEV-6 have also been found in wild boars in Japan [13]. In addition, HEV-7 and HEV-8 have been identified in dromedary camels in the Middle East and in Bactrian camels in China and Mongolia, respectively [13].

### 4.4. Acute Severe Hepatitis E

A systematic review and meta-analysis demonstrated that the pooled HEV-attributable proportion of viral-related ALF (*n* = 1312) was 40.9% (466/1138) (OR, 0.40; 95% CI: 0.28–0.52; *p* < 0.01), 30.6% (30/98) (OR, 0.30; 95% CI: 0.18–0.44; *p* = 0.15), and 60.5% (46/76) (OR, 0.61; 95% CI: 0.49–0.72; *p* = 0.90) among non-pregnant participants in India, China, and Bangladesh, respectively. However, a rate of 71.7% (485/676) (OR, 0.71; 95% CI: 0.62–0.79; *p* < 0.01) was recorded among pregnant Indian females [6]. The incubation period of HEV-ALF and factors leading to its progression were found to be 2–9 weeks and not known, respectively, by the authors, who also reported that the transplant-free survival rate of HEV-ALF was 55.1% (231/419) [93].

The prevalence of HEV-ALF has been shown to be relatively rare in viral ALF cases in Japan [94,95,96]. In Japan, when hepatitis occurs as a result of consumption of undercooked grilled pork, wild boar meat, or offal (including pig liver and intestines), HEV infection should be considered [13]. The routes of HEV infection have not yet been completely elucidated.

After the approval by Japan’s Health Insurance System of anti-HEV IgA antibody as a laboratory diagnostic tool for hepatitis E in 2011 [97], the number of hepatitis E cases has increased. As HEV-3 and HEV-4 may infect through transfusion, universal nucleic acid amplification testing-based blood-donor screening started in 2020 in order to prevent transfusion-transmitted HEV infection, revealing that asymptomatic indigenous HEV infection also exists in Japan [13]. HEV plays a role in the pathogenesis of non-A, non-B, and non-C ALF in developed countries such as Japan. Further studies are needed in this area; however, a positive test for HEV RNA is now established as the gold standard for the diagnosis of HEV infection [13].

In an Indian study, ACLF due to HEV infection was observed in 61.2% (74/121) of adult patients with cirrhosis [8]. HEV-ACLF has lower mortality (12.8% (5/39) vs. 33.3% (14/42)–54.5% (6/11) in other etiologies; *p* < 0.001) [98]. In a study from Bangladesh, positive tests for acute HEV infection were obtained in 21.7% (15/69) of ACLF patients [99]. In a Chinese study, patients infected with HEV-4 were found to be at high risk of developing ALF or ACLF [100]. Typical histopathological features of viral hepatitis may be absent in HEV-ACLF [101]. Thus, HEV infection is a trigger of ACLF [102,103,104,105].

Host factors (older age and genetic factors), viral factors (viral load, HEV genotype, nucleotide mutations in HEV RNA genomes), and other factors (coinfection with HIV, presence of chronic liver disease, existence of metabolic disease) have been identified as factors influencing the severity of HEV infection [13]. However, in most of the studies, HIV infection has not been found to affect HEV infection [106,107].

### 4.5. Vaccines and Challenges Facing Developers of Anti-HEV Drugs that Prevent HEV Infection

In China, HEV 239 (Hecolin) has been shown to be well-tolerated and effective in the prevention of hepatitis E [108,109,110,111]. Outside China, successful clinical trials of HEV 239 have also been reported in Bangladesh [110,112], the United States [111], and South Sudan [113,114]. In Japan, an HEV vaccine which could prevent the spread of HEV is still under development [13].

Ribavirin treatment is recommended in cases of severe acute hepatitis E or acute-on-chronic liver failure [13,14]. Japan’s Health Insurance System has not yet approved the use of ribavirin as a drug for the treatment of HEV infection [13]. Ribavirin is contraindicated for pregnant patients, patients with anemia, and patients with renal dysfunction. The development of more effective and safer drugs and the spread of vaccination for HEV infection are still needed [112].

Several research reports on anti-HEV drugs have been published. Nishiyama et al. reported that type III interferons (interferon λ1–3) could suppress HEV replication [115]. In another study, 2′-C-methylcytidine (2CMC)/ribavirin was found to exhibit a synergic effect against HEV replication [116]. Azithromycin and ritonavir have been shown to strongly inhibit HEV replication in vitro by the authors of [117]. The pan-cathepsin inhibitor K11777 has also been shown to suppress HEV infection [118]. Further studies in this line are still needed.

Gu et al. reported a wide variation in the quality of guidelines and primary recommendations regarding HEV, further stating that the evidence supporting the primary recommendations is currently of insufficient quality. As such, guideline developers and researchers should address these issues when updating and applying guidelines for the diagnosis and treatment of HEV infection [15]. We agree with their opinions to some extent.

Domestic HEV guidelines have also been published in China and India [15]. The Japan Agency for Medical Research and Development (AMED) HAV and HEV Study Group recently published two guidelines for HAV and HEV infections [12,13]. These reports are expected to aid in the diagnosis and treatment of acute and chronic HEV infection and to help prevent the progression of ALF and ACLF [12,13,15].

Chronic progression of HEV infection is typically observed in immunocompromised patients, and extrahepatic manifestation may be complicated with conditions such as prolonged hepatitis, glomerulonephritis and cryoglobulinemia, hematological disorders, and neuromuscular complications. In the control of HEV infection, HTAs, as well as DAAs, may be useful in disrupting host–HEV interactions by modulating the host cell pathways that are related to viral replication [119].

The Chinese consortium for the Study of Hepatitis E has reported the important findings of new results and better validation due to a larger cohort in HEV research [120,121,122]. Serum extracellular vesicle (EV)-derived argininosuccinate synthase 1 (ASS1) and serum exosome-derived aldehyde dehydrogenase 1 family member A1 (ALDH1A1) could effectively predict the occurrence and prognosis, respectively, of HEV-ALF [120,121]. It was reported that HEV infection leads to there being a large number of inflammatory cells in the pancreas and liver and that HEV infection affects the occurrence, development, and prognosis of acute pancreatitis [122].

Researchers from India have also greatly contributed to hepatitis E research [123,124,125,126,127,128,129,130,131]. Transmission routes of HEV infection and the clinical course of HEV-ALF were revealed by their larger number cohorts of HEV-infected patients with HEV infection and/or HEV-ALF [123,124,125,126,127,128]. Khan et al. reported that glucose and glutamine enhance HEV replication [129]. Kamar et al. reported that chronic hepatitis E occurred in organ-transplant recipients and that ribavirin is effective in treating chronic HEV infection [130,131]. Extremely careful attention should be paid to these matters.

In summary, we described the viral features of HEV, the present situation regarding HEV infection in representative Asian countries, and the development of anti-HEV drugs and HEV vaccines. It is important for medical researchers to understand that HEV is one of the causes of ALF and ACLF and that the clinical features of HEV infection depend on the HEV genotype.

The different roles played by HAV and HEV in both ALF and ACLF are shown in Table 1. HEV-3 and HEV-4 infection could lead to chronic hepatitis or cirrhosis, especially in immunocompromised patients. Alcohol-associated liver disease, chronic HBV infection, or chronic HCV infection are major causes of chronic liver diseases or cirrhosis in ACLF patients. Attention should be paid to HEV-3 or HEV-4 infection as a cause of chronic liver disease and to HEV infection as a cause of acute insult in ACLF patients in Asian countries. Although HEV infection leads to a mortality of up to approximately 30% in pregnant women in the third trimester [119,132], it is possible that host immunity may be associated with the severity of HEV diseases. The interplay between HEV infection and host cell pattern recognition receptors, involving the innate immune response and virus-mediated immune evasion, may play an important role in hepatic and extrahepatic manifestations such as acute pancreatitis and neurologic disorders [133,134].

## 5. Coinfection with HAV and HEV

Of the 1807 specimens processed from the patients with acute viral hepatitis at a tertiary care hospital in Western India, 6.7% (120/1807), 8.5% (154/1807), and 0.6% (11/1807) were positive for only IgM anti-HAV antibodies, only IgM anti-HEV antibodies, and both antibodies, respectively [135]. All patients coinfected with HAV and HEV had deranged liver function tests indicating more severe disease. Dual HAV and HEV infection could further exacerbate liver damage, leading to ALF with a higher mortality rate than that of infection with either virus along [136]; notably, the relative risk of ALF development was reported to be 8.5 times higher in HAV–HEV coinfection compared with HAV or HEV mono-infection [137]. Although both viruses have a similar pathogenesis and similar transmission routes, coinfection with HAV and HEV may result in more severe disease than may mono-infection.

## 6. Discussion

Recommendations for the diagnosis, treatment, and prevention of HEV produced by the World Health Organization (WHO), EASL, AMED (Japan), and the Indian National Association for the Study of the Liver (INASL) have been published [13,14,138,139]. This literature comprehensively documents the pathogenesis, presentation, and prognosis associated with HAV and HEV infection.

We report the prevalence or incidence of ALF and ACLF caused by HAV or HEV infection in the representative Asian countries in Table 2. Unfortunately, most countries in Asia did not report the national prevalence or incidence of severe liver diseases [140,141]. Further studies will be needed.

At present, universal mass vaccination against hepatitis A and hepatitis E are available in a limited number of Asian countries [108,109,110,111,142]. We encourage other countries to start mass vaccination programs against HAV and HEV. There are various treatments for ALF and ACLF in Asian countries, and they need to be developed further in Asia [1,141].

## 7. Conclusions

HAV and HEV are important infections that can cause severe liver diseases globally. We reviewed the role of HAV and HEV in ALF and ACLF in Asian countries. Hepatitis A should be recognized as one of the sexually transmitted infections, especially among MSM. HAV genotype IIIA infection seems to present a more severe clinical manifestation. Acute HEV-1 infection is associated with ALF in pregnant women in India. HEV-4, rather than HEV-3, was found in patients with severe hepatitis in Japan. HEV also plays a role as a cause of acute insult and/or chronic liver disease in immunocompromised patients with ACLF. Further studies are needed to develop vaccines and antivirals against HAV and HEV infections. Despite the limitations in the recording of cases and the extent of specific vaccinations, multidisciplinary cooperation, involving hepatologists, virologists, experts in public health, infectious disease specialists, pharmacists, etc., could improve the treatment of HAV and HEV infection.

## Figures and Tables

**Table 1:** Different roles of hepatitis A and E viruses in acute severe hepatitis.

**Item**	**ALF**	**ACLF**
Background liver	No liver diseases	Chronic liver diseases or cirrhosis
**Acute insults**		
HAV	Yes	Yes
HEV	Yes	Yes
**Chronic liver diseases**		
HAV	No	No
HEV	No	Yes (HEV-3 or HEV-4 in immunocompromised patients)

ALF—acute liver failure; ACLF—acute-on-chronic liver failure; HAV—hepatitis A virus; HEV—hepatitis E virus.

**Table 2:** Prevalence or incidence of acute severe hepatitis caused by hepatitis A virus (HAV) and hepatitis E virus (HEV) infection in the representative Asian countries. (A) Acute liver failure (ALF) and (B) acute-on-chronic liver failure (ACLF).

**(A)**			
**Country**	**Prevalence %** **(*n*/Total Patients)**	**Ref.**	**Note**
China	HEV (non-pregnant), 30.6% (30/98)	[6]	"Total patients" means all viral-related ALF patients.
Bangladesh	HEV (non-pregnant), 60.5% (46/76)	[6]	"Total patients" means all viral-related ALF patients.
India	HAV, 44.2% (81/183)	[58]	"Total patients" means all ALF patients.
India	HEV (non-pregnant), 40.9% (466/1138)	[6]	"Total patients" means all viral-related ALF patients.
India	HEV (pregnant women), 71.7% (485/676)	[6]	"Total patients" means all viral-related ALF patients.
Japan	HAV, 1.5% (77/4994)	[57,140]	"Total patients" means all acute hepatitis A patients (1999–2015).
Japan	HEV, 3.1% (23/742)	[4,140]	"Total patients" means all acute hepatitis E patients (2010–2015).
South Korea	HAV, 0.5% (3/595)	[35]	"Total patients" means all acute hepatitis A patients.
**(B)**			
**Country**	**Prevalence %** **(*n*/Total Patients)**	**Ref.**	**Note**
APASL	HAV or HEV, 3.1% (118/369)	[141]	"Total patients" means all participants.
Bangladesh	HEV, 21.7% (15/69)	[99]	"Total patients" means all ACLF patients.
India	HAV, 27.2% (33/121)	[8]	"Total patients" means all ACLF patients.
India	HEV, 61.2% (74/121)	[8]	"Total patients" means all ACLF patients.

APASL—Asian Pacific Association for the Study of the Liver.

## Data Availability

Not applicable.
